# Biallelic deleterious germline *SH2B3* variants cause a novel syndrome of myeloproliferation and multi‐organ autoimmunity

**DOI:** 10.1002/jha2.698

**Published:** 2023-04-30

**Authors:** Piers Blombery, Vahid Pazhakh, Adriana S. Albuquerque, Jesmeen Maimaris, Lingge Tu, Brenda Briones Miranda, Florence Evans, Ella R. Thompson, Ben Carpenter, Ian Proctor, Julie A. Curtin, Jonathan Lambert, Siobhan O. Burns, Graham J. Lieschke

**Affiliations:** ^1^ Clinical Haematology Peter MacCallum Cancer Centre/Royal Melbourne Hospital Melbourne Victoria Australia; ^2^ University of Melbourne Melbourne Victoria Australia; ^3^ Australian Regenerative Medicine Institute Monash University Clayton Victoria Australia; ^4^ Institute of Immunity and Transplantation University College London London UK; ^5^ Department of Immunology Royal Free London NHS Foundation Trust London UK; ^6^ Department of Haematology University College London Hospitals NHS Foundation Trust London UK; ^7^ Haematology Department Children's Hospital at Westmead Westmead New South Wales Australia; ^8^ Department of Haematology UCL Cancer Institute University College London London UK

**Keywords:** genetics, molecular diagnosis, myeloid function and development

## Abstract

*SH2B3* is a negative regulator of multiple cytokine receptor signalling pathways in haematopoietic tissue. To date, a single kindred has been described with germline biallelic loss‐of‐function *SH2B3* variants characterized by early onset developmental delay, hepatosplenomegaly and autoimmune thyroiditis/hepatitis. Herein, we described two further unrelated kindreds with germline biallelic loss‐of‐function *SH2B3* variants that show striking phenotypic similarity to each other as well as to the previous kindred of myeloproliferation and multi‐organ autoimmunity. One proband also suffered severe thrombotic complications. CRISPR‐Cas9 gene editing of zebrafish *sh2b3* created assorted deleterious variants in F0 crispants, which manifest significantly increased number of macrophages and thrombocytes, partially replicating the human phenotype. Treatment of the *sh2b3* crispant fish with ruxolitinib intercepted this myeloproliferative phenotype. Skin‐derived fibroblasts from one patient demonstrated increased phosphorylation of JAK2 and STAT5 after stimulation with IL‐3, GH, GM‐CSF and EPO compared to healthy controls. In conclusion, these additional probands and functional data in combination with the previous kindred provide sufficient evidence for biallelic homozygous deleterious variants in *SH2B3* to be considered a valid gene‐disease association for a clinical syndrome of bone marrow myeloproliferation and multi‐organ autoimmune manifestations.

## 1 INTRODUCTION

*SH2B3* (SH2B adapter protein 3) is a negative regulator of multiple cytokine receptor signalling pathways in hematopoietic tissue including Signal transducer and activator 5 (STAT5), Protein kinase B (PKB/AKT) and Mitogen‐activated protein kinase (MAPK) [1, 2]. Acquired deleterious *SH2B3* variants resulting in increased Janus kinase/signal transducer and activator of transcription (JAK/STAT) signalling are observed in approximately 5% of chronic phase myeloproliferative neoplasms (MPN) either in the presence or absence of canonical MPN driver variants (e.g. *JAK2* Val617Phe) [3, 4]. In contrast to the acquired setting, the clinical implications and any phenotype associated with deleterious germline variants in *SH2B3* are less clear. A single kindred has been previously described with two affected probands carrying homozygous frameshift *SH2B3* variants who presented at a young age with hepatosplenomegaly, autoimmune hepatitis, developmental delay and autoimmune thyroiditis [5]. One of these probands also developed B‐acute lymphoblastic leukaemia [6, 7]. Despite this initial important description, there is currently insufficient evidence to definitively establish a clinically valid gene‐disease pair resulting from germline deleterious *SH2B3* variants according to formal criteria [8]. Moreover, the potential breadth of any phenotype is currently unknown. Herein, we provide clinical and functional data on two further unrelated kindred that harbor biallelic deleterious germline *SH2B3* variants and establish a novel germline syndrome with the clinical phenotype of myeloproliferation and multi‐organ autoimmunity.

## 2 METHODS

### 2.1 Patient identification and genomic investigation

Two unrelated patients were identified from Peter MacCallum Cancer Centre (PMCC, Melbourne, Australia) and University College London Hospital (UCLH, London, United Kingdom). Patient 1 underwent next generation sequencing (NGS) using a targeted panel as previously described [9], patient 2 underwent whole genome sequencing (WGS) as part of as part of the PID domain of the United Kingdom NIHR BioResource—Rare Diseases program [10].

### 2.2 Zebrafish sh2b3 loss‐of‐function models

CRISPR/Cas9 mutagenesis of zebrafish *sh2b3* was performed as previously described [11, 12]. Briefly, three guide RNAs (gRNAs) targeting *sh2b3* exon 1 (Table S1) were microinjected together in 1‐cell embryos of reporter lines marking hematopoietic stem cells, myeloid cells and thrombocytes and quantified in F0 knockdown crispants by manual counting of fluorescent cells. Ruxolitinib was administered by immersion at 4 μM, renewed daily, for up to 5 dpf (see Supplementary Methods).

### 2.3 Assessment of signalling from skin fibroblasts

Cultured skin‐derived fibroblasts from patient 2 were treated for 15 min with interleukin‐3 (IL‐3, 20 ng/ml and/or 200 ng/ml), growth hormone (GH, 100 ng/ml and/or 500 ng/ml), granulocyte‐macophage colony‐stimulating factor (GM‐CSF, 10 ng/ml and/or 100 ng/ml) or erythropoietin (EPO, 15 ng/ml). Native and phosphorylated signalling proteins were then assessed by immunoblotting and flow cytometry (see Supplementary Methods).

## 3 RESULTS AND DISCUSSION

**FIGURE 1:**
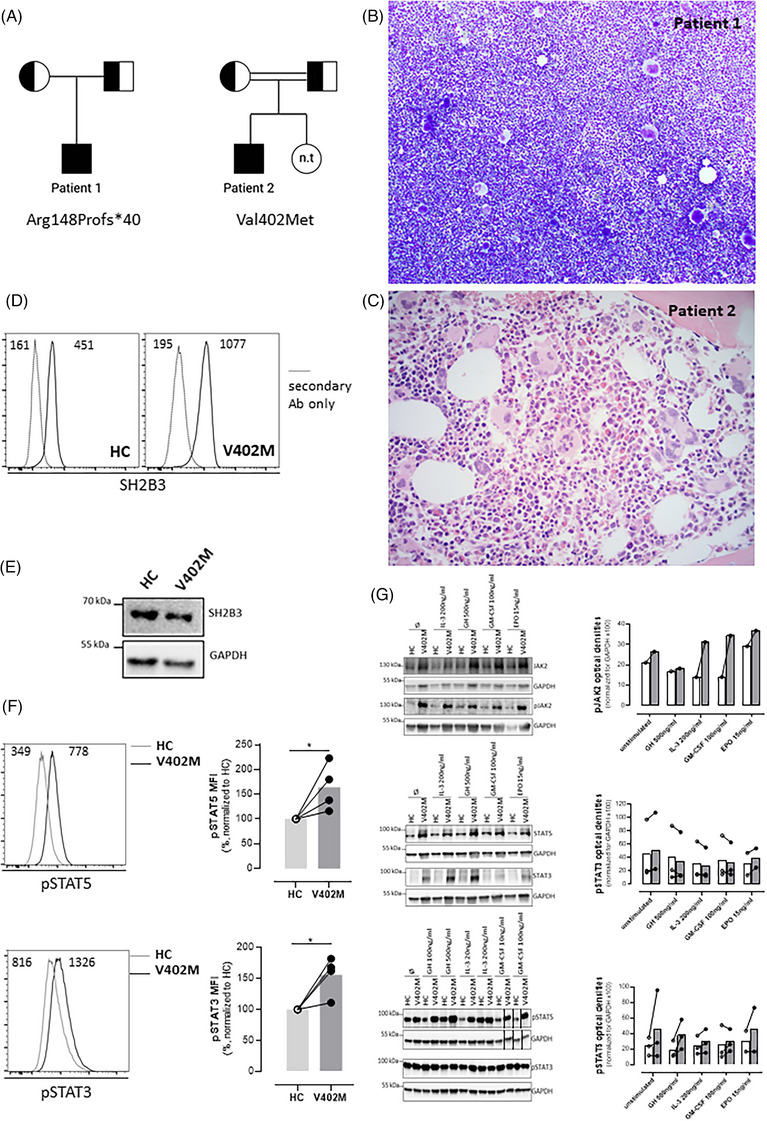
Clinicopathological features of patients with biallelic loss of function variants in *SH2B3*. (A) Pedigree for patients 1 and 2; half‐filled squares and circles indicate carrier status for *SH2B3* variants for males and females, respectively. Fully filled squares indicate homozygous *SH2B3*. n.t. – not tested. Double line indicates consanguinity. (B) Bone marrow aspirate from patient 1 demonstrating hypercellular marrow with megakaryocytic hyperplasia and morphological atypia (×100). (C) Bone marrow trephine biopsy from patient 2 demonstrated megakaryocytic hyperplasia and atypia (haematoxylin and eosin (H&E), ×400). (D) SH2B3 expression assessed intracellularly by flow cytometry on skin‐derived fibroblasts using a mouse anti‐human SH2B3 antibody followed by goat anti‐mouse IgG‐AlexaFluor488 staining after fixation and permeabilization using the BD Cytofix/CytoPerm buffers. Immunoreactive SH2B3 expression is present in wildtype healthy control (HC) and proband (V402M) cells. Negative control is secondary antibody (Ab) only. Numbers inside panels represent mean fluorescence intensity (MFI). (E) Western blot showing normal expression of SH2B3 in V402M skin‐derived fibroblasts; GADPH loading control. (F) Basal STAT‐5 and STAT‐3 phosphorylation was evaluated on skin‐derived fibroblasts by Phosflow assay using BD Phosflow Fix I and Perm III buffers according to manufacturer instructions (BD Biosciences). Histograms on the left show a representative example and graphs on the right the normalised mean values for four experiments. Numbers inside histograms represent mean fluorescence intensity (MFI). pSTAT3 and pSTAT5 levels were significantly elevated relative to control in the proband (V402M) samples (*indicates *p* < 0.05, Mann–Whitney test). (G) Increased phosphorylation of JAK2 and STAT5 in SH2B3‐V402M. Skin‐derived fibroblasts were obtained from patient 2 and from a healthy control were assessed for SH2B3, pJAK2, pSTAT3 and pSTAT5 protein expression by immunoblotting before and after 15‐min stimulation with the indicated ligands (optical density for experiment and replicates provided in histogram). Black lines next to samples in lanes 13 and 14 (pSTAT5 and GAPDH) indicate where images have been moved in the figure to allow comparison between healthy control versus patient. Graphs on the right show quantitation of multiple immunoblots with lines connecting paired samples. EPO, erythropoietin; GH, growth hormone; GM‐CSF, granulocyte‐macrophage colony‐stimulating factor; HC, healthy control; IL‐3, interleukin‐3.

Patient 1 was a male who presented with splenomegaly, thrombocytosis, neutrophilia and a leukoerythroblastic blood film at 3 months of age (Figure 1A). A bone marrow biopsy demonstrated myeloid hyperplasia with megakaryocytic hyperplasia and atypia (Figure 1B). The patient developed alopecia areata at age 2 and autoimmune hypothyroidism at age 6. He was otherwise developmentally normal without any detected liver abnormalities or thrombotic episodes. His splenomegaly and neutrophil count gradually improved throughout life; however, his platelet count remained markedly elevated (∼1000 × 10^9^/L at 18 years of age). At age 18, the patient underwent investigation with an NGS panel as previously described^9^ and a homozygous frameshift variant was detected in *SH2B3* (NM_005475.3:c.441_468del; p.(Arg148Profs*40)). Both parents (with no history of cytoses or autoimmune disease) were found to be heterozygous carriers of this same variant (Figure 1A)

Patient 2 was a male born to consanguineous Iranian parents (Figure 1A) who was noted shortly after birth to have isolated splenomegaly for which no cause was identified. A persistent thrombocytosis was noted throughout life ranging from 550–780 × 10^9^/L. At age 12, he was diagnosed with autoimmune hypothyroidism and Raynaud syndrome. At 17 years old, he developed acute hepatitis with liver biopsy features consistent with autoimmune hepatitis. A bone marrow biopsy at this stage demonstrated a mildly hypercellular marrow with megakaryocytic hyperplasia and atypia (Figure 1C). In addition, he was also noted to be hyperglycemic and was diagnosed with autoimmune diabetes mellitus and commenced on insulin. His liver function tests improved with prednisolone and tacrolimus; however, he presented 2 months later with left‐sided weakness and on investigation was found to have a right‐sided middle cerebral artery territory infarct with a right carotid artery thrombus.

The patient and his parents underwent trio WGS testing and a homozygous missense variant in *SH2B3* (c.1204G>A; p.(Val402Met)) was detected in the patient with both parents found to be heterozygous carriers (Figure 1A). The *SH2B3* Val402Met has been observed multiple times in hematological malignancy (https://cancer.sanger.ac.uk/cosmic) as an acquired variant, occurs in the SH2 domain (which is a recurrent site of acquired mutations in myeloproliferative neoplasms^4^) and has been previously been shown to have reduced negative regulating activity in vitro [13]. Skin‐derived fibroblasts were obtained from the patient which demonstrated SH2B3 protein expression (Figure 1D, E), a basal increase in pSTAT5 and pSTAT3 by phosphoflow compared to healthy cells (Figure 1F). In addition, increased pJAK2 and pSTAT5 by immunoblotting after stimulation with IL‐3, GH, GM‐CSF and EPO compared to healthy controls was demonstrated by immunoblotting (Figure 1G).

**FIGURE 2:**
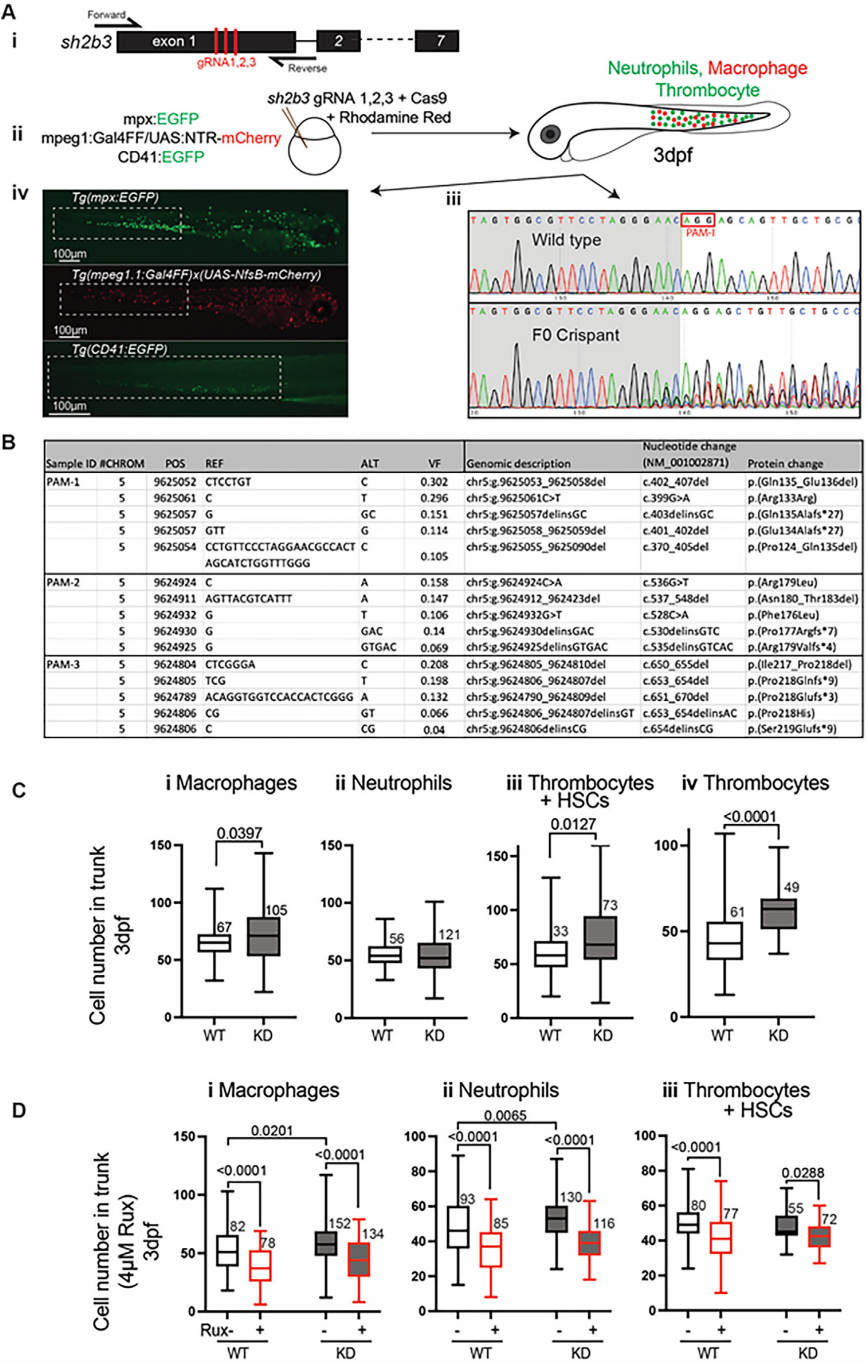
Zebrafish *sh2b3* loss‐of‐function model. (Ai) CRISPR/Cas9‐mediated mutagenesis to truncate the *sh2b3* at exon 1. Red bars indicate the target sites of three designed gRNAs. (Aii) Microinjection of 3x gRNAs into one‐cell stage zebrafish embryo along with Cas9 protein and rhodamine. (Aiii) Sanger sequencing of *sh2b3* locus in wild type (top) and F0 crispant (bottom) zebrafish embryos indicating on‐target gene disruption at gRNA‐1 target site (as an example). The red rectangle indicates the PAM sequence for the gRNA‐1. (Aiv) Respectively, EGFP and mCherry expressing neutrophils and macrophages in *Tg(mpx:EGFP)* and *Tg(mpeg1.1:Gal4FF/UAS:NfsB‐mCherry)*, and EGFP expressing cells (HSCs and thrombocytes) in *Tg (CD41:EGFP)* 3dpf zebrafish embryos. Dashed white rectangles mark regions in which cells have been quantified. (B) NGS result showing the top 5 most common variants at each gRNA target site. (C) Quantification of macrophages (*mpeg1:mCherry* ^positive^), neutrophils (*mpx:EGFP* ^positive^), HSCs and thrombocytes (*CD41:EGFP* ^positive^) and thrombocytes (*mpl:EGFP* ^positive^) in the tail region of 3 dpf *sh2b3* knockdown (F0 crispants) compared to wildtype embryos. (D) Quantification of macrophages, neutrophils and *CD41:EGFP* ^positive^ cells in *sh2b3* knockdown and wildtype embryos in the presence or absence of 4 μM ruxolitinib. HSCs, hematopoietic stem cells; gRNA, guide RNA; dpf, days post fertilization; KD, knockdown; PAM, protospacer adjacent motif; VF, variant frequency; Rux, Ruxolitinib; WT, wild‐type. Box and whisker plots (range, 25th and 75th percentile, median) for *n* embryos (*n* values near box) pooled from three biologically independent experiments. *p*‐Values from unpaired *T*‐test (C) and one‐way ANOVA with Tukey's multiple comparison test (D); *p*‐values shown only for significant differences.

To model the *SH2B3* Arg148Profs*40 variant from patient 1, CRISPR‐Cas9 gene editing [11, 14] was targeted to the analogous region of zebrafish *sh2b3* to create assorted deleterious variants in F0 crispants (Figure 2A). Sanger sequencing confirmed on‐target editing with NGS demonstrating a mixture of in‐frame, missense and frameshift variants (Figure 2B). F0 crispants had a significantly increased number of macrophages and thrombocytes, replicating the patient phenotype (Figure 2C). Treatment of the *sh2b3* crispant fish with the JAK inhibitor ruxolitinib (previously demonstrated to be active against zebrafish JAK2 [15]) intercepted the myeloproliferative phenotype (Figure 2D). The myeloproliferative phenotype was not observed in stable deletion mutants, likely reflecting genetic compensation not present in crispants (Figure S1) [16].

The clinical phenotype of these two patients demonstrates remarkable similarity to each other as well as the previously reported kindred [5]. The haematopoietic manifestations were dominated by myeloproliferative features consistent with the established role of *SH2B3* in negatively regulating signalling from the erythropoietin and thrombopoietin receptors [1, 17]. Moreover, we provide two independent lines of evidence linking *SH2B3* dysfunction to this disease phenotype by (1) reproducing this myeloproliferation by CRISPR‐Cas9 mediated disruption of zebrafish *sh2b3* and (2) demonstrating increased phosphorylation of JAK2, STAT5, and STAT3. Notably, *Sh2b3* knockout models in both mouse and rat generated by others show similar myeloproliferative features as well as splenomegaly [18, 19]. Importantly, despite evidence of generalized myeloproliferation, neither of our patients had clinicopathological evidence of a haematological malignancy (either myeloid or lymphoid lineage) during follow‐up.

Both of our patients developed clinically significant extra‐haematopoietic multi‐organ autoimmune manifestations throughout life including autoimmune hypothyroidism, autoimmune hepatitis, alopecia areata and autoimmune diabetes mellitus. Whilst prominent autoimmune manifestations have not been noted in model organisms to date (including our zebrafish model) [18, 19], the spectrum of autoimmunity observed in our patients closely mirrors the established increased risk of autoimmune phenomena associated with common loss‐of‐function *SH2B3* germline polymorphisms established through genome wide association studies and serves to further support the validity of these increased risks in the heterozygous state [20, 21, 22, 23, 24, 25].

In conclusion, we propose that these additional probands and functional data in combination with the previous kindred provide sufficient evidence for biallelic deleterious variants in *SH2B3* to be considered a valid gene‐disease association for a clinical syndrome of bone marrow myeloproliferation and multi‐organ autoimmune manifestations. Descriptions of further affected individuals will be required to fully understand the breadth of the phenotype. Moreover, our data support ruxolitinib as a potential therapeutic option for patients with this rare immune dysregulatory syndrome.

## AUTHOR CONTRIBUTIONS

PB conceived of the study, managed the patients, analysed data and wrote the manuscript; VP, AA, LT, BBM, FE, ERT performed experiments and analysed data; JM, BC, JL, JAC, SOB managed the patients and analysed data; IP analysed histopathology investigations; GJL established and supervised zebrafish modelling, analysed data and wrote the manuscript. All authors contributed to and approved of the final version of the manuscript.

### CONFLICT OF INTEREST STATEMENT

P.B. consulted for, advised, or received honoraria from Adaptive Biotechnologies, AstraZeneca, and Servier; S.B. has received personal fees or travel expenses from Immunodeficiency Canada/IAACI, CSL Behring, Baxalta US Inc., GSK and Biotest; G.J.L. has consulted for CSL Behring; J.L. has served on advisory boards for Kite/Gilead and received honoraria from Takeda.

### ETHICS STATEMENT

Informed written consent was obtained in accordance with the Declaration of Helsinki and approval from the local ethics committee (reference number: 04/Q0501/119).

## Supporting information

Supporting Information
