# Supplementary material for: Biallelic deleterious germline SH2B3 variants cause a novel syndrome of myeloproliferation and multi‐organ autoimmunity
Source: EJHaem. 2023 Apr 30;4(2):463–9. doi: 10.1002/jha2.698 (PMC10188477; doi:10.1002/jha2.698)
Supplement: Supplementary file 1 — Supporting Information [file JHA2-4-463-s001.pdf]

Supplementary Data for Blombery P et al. Biallelic deleterious germline *SH2B3* variants cause a novel clinical syndrome of myeloproliferation and multi-organ autoimmunity

Supplementary table 1. Guide RNAs (gRNAs) targeting *sh2b3* exon 1

Guide RNA sequence	
gRNA-1	UAGUGGCGUUCCUAGGGAAC
gRNA-2	UGAGUUACGUCAUUUCGGGA
gRNA-3	AGGGACGAGUUGAUCCCGAG

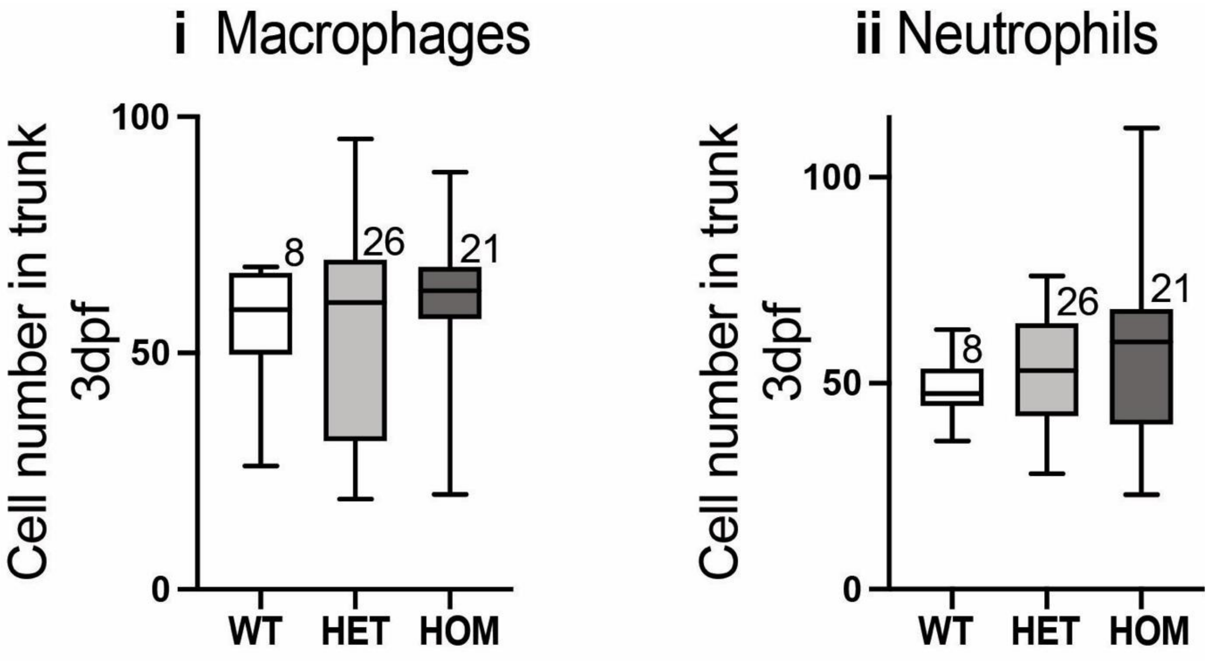

Supplementary Figure 1. Absence of myeloproliferative phenotype in mutant zebrafish embryos carrying *sh2b3* loss-of-function alleles (compound heterozygous) Quantification of neutrophils and macrophages in F2 zebrafish embryos carrying one or two *sh2b3* loss-of-function alleles (compound heterozygous genotypes as given below) and wildtype embryos. dpf, days post fertilization, WT: wild type, HET: heterozygous, HOM: homozygous. 1-way ANOVA with Tukey's correction for multiple

comparisons revealed no significant differences. Box and whisker plots (range, 25th and 75th percentile, median) for n embryos (n values near box) pooled from 3 biologically independent experiments. Compound heterozygous alleles in each experiment:

Experiment No.	HGVSg	HGVSc	HGVSp
1	Chr5:g.9,624,806_9,625,061del	c.399_654del	p.Glu134Valfs*6
1,2	Chr5:g.9,625,057_9,625,061delinsAGTAGG	c.399_403delinsCCTACT	p.Arg133Serfs*29
2	Chr5:g.9,624,808_9,625,058delinsTAACTCCTGT TGC	c.402_652delinsGCAACAGGA GTTA	p.Glu136Glnfs*10
3	Chr5:g.9,624,927_9,625,059del	c.401_533del	p.Glu134Alafs*5
3	Chr5:g.9,624,951_9,625,069del	c.391_509del	p.Val131Serfs*11
3	Chr5:g.9,624,926_9,625,061del	c.400_535del	p.Glu134Alafs*6
3	Chr5:g.9,625,060_9,625,069del	c.391_400del	p.Val131Asnfs*49

Supplementary Methods

Zebrafish sh2b3 loss-of-function models

Zebrafish (*Danio rerio*) experiments were conducted in accordance with a Monash University Animal Ethics Committee approved protocol (Project ID: 17270). Strains used were: a combination transgenic reporter line *Tg(mpeg1.1:Gal4FF)^gl25* [1] x *Tg(UAS-E1b:Eco.NfsB-mCherry)^c264* [2] x *Tg(mpx:EGFP)^i114* [3], in which mCherry and EGFP are expressed in macrophages and neutrophils respectively; *Tg(CD41:EGFP)^la2* [4] with EGFP^*low* expressing hematopoietic stem cells and EGFP^*high* expressing thrombocytes; and *Tg(mpl:EGFP)* expressing EGFP in thrombocytes, generated in-house by reinjection of the construct made and generously provided by Y. Zhang[5].

CRISPR/Cas9 mediated mutagenesis of zebrafish *sh2b3* was performed as previously described[6,7]. Briefly, three optimal guide RNAs (gRNA) targeting *sh2b3* exon 1 (which encodes the linker between the first and second protein functional domains) were microinjected together in 1-cell embryos. On-target gene editing was confirmed by performing Sanger sequencing and next generation sequencing (NGS) of randomly selected crispant embryos, as previously described[8]. For gRNA sequences, see Supplementary table 1.

Adult F0 crispants were outcrossed to *sh2b3* wildtype fish to segregate mutant alleles in F1 offspring. Compound heterozygous F2 animals were generated by incrossing heterozygous F1 zebrafish. Haematopoietic cells were quantified in F0 knockdown crispant and F2 stable mutant zebrafish embryos by manual counting of fluorescent expressing cells in images taken on Olympus MXV10 microscope fitted with an Olympus DP72 camera and CellSens software (Version 1.11).

Ruxolitinib (STEMCELL Technologies) was dissolved in DMSO at a stock concentration of 10 mM (stored at -20 °C) and administered by immersion at final concentration of 4 µM, renewed daily, from immediately after injection to 5 dpf.

Cytokine signalling assessment of skin fibroblasts

Skin-derived fibroblasts were obtained from patient 2 and from a healthy control. Fibroblasts were cultured in DMEM-GlutaMAX (Gibco) supplemented with 10% FCS (Gibco) and 1% penicillin-streptomycin (Sigma-Aldrich). Cells were left untreated or were treated for 15min with IL-3 (20 ng/ml and/or 200ng/ml), growth hormone (GH, 100 ng/ml and/or 500 ng/ml), granulocyte-macrophage colony stimulating factor (GM-CSF, 10 ng/ml and/or 100 ng/ml) or erythropoietin (EPO, 15 ng/ml). Informed written consent was obtained in accordance with the Declaration of Helsinki and approval from the local ethics committee (reference number 04/Q0501/119).

Western blot analysis

Cells were collected, washed with cold PBS, and lysed in RIPA (Sigma-Aldrich) lysis buffer containing 1X protease inhibitor cocktail (Roche) for 30min on ice. Cell lysates were centrifuged at 12000rpm for 20min at 4°C and supernatant was collected. Samples were subjected to SDS-polyacrylamide gel electrophoresis (10% Mini-Protean TGX Precast Protein Gels, Biorad) and transferred to nitrocellulose membranes (Biorad). After blocking with 5% BSA for 1h at room temperature, the membranes were probed with the following primary (overnight at 4°C) and HPR-conjugated secondary antibodies (1h at

room temperature): rabbit anti-phospho-STAT5A/B (Tyr694, Santa Cruz Biotechnology), rabbit anti-STAT5 (clone C-17, Santa Cruz Biotechnology), rabbit anti-phospho-STAT3 (clone D3A7, Cell Signalling Technology), rabbit anti-STAT3 (clone 79D7, Cell Signalling Technology), rabbit anti-phospho-JAK2 (clone D4A8, Cell Signalling Technology), rabbit anti-JAK2 (clone D2E12, Cell Signalling Technology), rabbit anti-GAPDH (clone D16H11, Cell Signalling Technology), mouse anti-SH2B3 (clone A12, Santa Cruz Biotechnology), anti-rabbit-IgG-HRP (Cell Signalling Technology) and anti-mouse-IgG-HRP (Cell Signalling Technology). The proteins were detected with SuperSignal West Pico Plus chemiluminescent substrate (ThemoFisherScientific) using the ChemiDoc Imaging System (Biorad).

Intracellular expression of SH2B3 by flow cytometry

SH2B3 expression was detected intracellularly by flow cytometry on skin-derived fibroblasts. Cells were fixed for 20min using BD Cytofix buffer (BD Biosciences) and permeabilized for 30min on ice using BD Cytoperm buffer (BD Biosciences). Staining was performed using an unconjugated mouse anti-human SH2B3 antibody (clone A12, Santa Cruz Biotechnology) followed by indirect staining with an anti-mouse-IgG-AlexaFluor488 (ThermoFisher). At least 100000 events were acquired on a BD LSRFortessa flow cytometer (BD Biosciences). Data was analysed using FlowJo software (Tree Star) after doublet exclusion. Results are presented as mean fluorescence intensity (MFI).

Phosphoflow assay

Fibroblasts were fixed for 10min at 37°C using Fix I buffer (BD Biosciences) and then permeabilized for 30min at 4°C using Fix III buffer (BD Biosciences). Cells were stained for 30min at room temperature using an anti-human pSTAT5 (clone 47/Stat5 pY694, BD Biosciences) and anti-human-pSTAT3 antibodies (clone 4/P-STAT3 pY705, BD Biosciences) and washed. At least 100000 events were acquired on a BD LSRFortessa flow cytometer (BD Biosciences). Data was analysed using FlowJo

software (Tree Star) after doublet exclusion. Results are presented as mean fluorescence intensity (MFI).

Statistical analysis

Statistical analysis was performed using GraphPad Prism (v9.3.1). Data were compared using two-way t-tests, Mann-Whitney tests, and 1-way ANOVA with Tukey's multiple comparison test. P values <0.05 were considered significant.

1. Ellett F, Pase L, Hayman JW, Andrianopoulos A, Lieschke GJ. mpeg1 promoter transgenes direct macrophage-lineage expression in zebrafish. *Blood*. 2011;117(4):e49-56.
2. Davison JM, Akitake CM, Goll MG, et al. Transactivation from Gal4-VP16 transgenic insertions for tissue-specific cell labeling and ablation in zebrafish. *Dev Biol*. 2007;304(2):811-824.
3. Renshaw SA, Loynes CA, Trushell DM, Elworthy S, Ingham PW, Whyte MK. A transgenic zebrafish model of neutrophilic inflammation. *Blood*. 2006;108(13):3976-3978.
4. Lin HF, Traver D, Zhu H, et al. Analysis of thrombocyte development in CD41-GFP transgenic zebrafish. *Blood*. 2005;106(12):3803-3810.
5. Lin Q, Zhang Y, Zhou R, et al. Establishment of a congenital amegakaryocytic thrombocytopenia model and a thrombocyte-specific reporter line in zebrafish. *Leukemia*. 2017;31(5):1206-1216.
6. Pazhakh V, Clark S, Keightley MC, Lieschke GJ. A GCSFR/CSF3R zebrafish mutant models the persistent basal neutrophil deficiency of severe congenital neutropenia. *Sci Rep*. 2017;7:44455.
7. Gagnon JA, Valen E, Thyme SB, et al. Efficient mutagenesis by Cas9 protein-mediated oligonucleotide insertion and large-scale assessment of single-guide RNAs. *PLoS One*. 2014;9(5):e98186.
8. Isiaku AI, Zhang Z, Pazhakh V, et al. Transient, flexible gene editing in zebrafish neutrophils and macrophages for determination of cell-autonomous functions. *Dis Model Mech*. 2021;14(7).
